# Self-reported and objectively assessed knowledge of evidence-based practice terminology among healthcare students: A cross-sectional study

**DOI:** 10.1371/journal.pone.0200313

**Published:** 2018-07-12

**Authors:** Anne Kristin Snibsøer, Donna Ciliska, Jennifer Yost, Birgitte Graverholt, Monica Wammen Nortvedt, Trond Riise, Birgitte Espehaug

**Affiliations:** 1 Centre for Evidence-Based Practice, Faculty of Health and Social Sciences, Western Norway University of Applied Sciences, Bergen, Norway; 2 School of Nursing, Faculty of Health Sciences, McMaster University, Hamilton, Ontario, Canada; 3 College of Nursing, Villanova University College of Nursing, Villanova, Pennsylvania, United States of America; 4 Accident & Emergency Department, Bergen Municipality, Bergen, Norway; 5 Department of Global Public Health and Primary Care, University of Bergen, Bergen, Norway; University of Antwerp, BELGIUM

## Abstract

**Background:**

Self-reported scales and objective measurement tools are used to evaluate self-perceived and objective knowledge of evidence-based practice (EBP). Agreement between self-perceived and objective knowledge of EBP terminology has not been widely investigated among healthcare students.

**Aim:**

The aim of this study was to examine agreement between self-reported and objectively assessed knowledge of EBP terminology among healthcare students. A secondary objective was to explore this agreement between students with different levels of EBP exposure.

**Methods:**

Students in various healthcare disciplines and at different academic levels from Norway (n = 336) and Canada (n = 154) were invited to answer the Terminology domain items of the Evidence-Based Practice Profile (EBP^2^) questionnaire (self-reported), an additional item of ‘evidence based practice’ and six random open-ended questions (objective). The open-ended questions were scored on a five-level scoring rubric. Interrater agreement between self-reported and objective items was investigated with weighted kappa (K_w_). Intraclass correlation coefficient (ICC) was used to estimate overall agreement.

**Results:**

Mean self-reported scores varied across items from 1.99 (‘forest plot’) to 4.33 (‘evidence-based practice’). Mean assessed open-ended answers varied from 1.23 (‘publication bias’) to 2.74 (‘evidence-based practice’). For all items, mean self-reported knowledge was higher than that assessed from open-ended answers (p<0.001). Interrater agreement between self-reported and assessed open-ended items varied (K_w_ = 0.04–0.69). The overall agreement for the EBP^2^ Terminology domain was poor (ICC = 0.29). The self-reported EBP^2^ Terminology domain discriminated between levels of EBP exposure.

**Conclusion:**

An overall low agreement was found between healthcare students’ self-reported and objectively assessed knowledge of EBP terminology. As a measurement tool, the EBP^2^ Terminology scale may be useful to differentiate between levels of EBP exposure. When using the scale as a discriminatory tool, for the purpose of academic promotion or clinical certification, users should be aware that self-ratings would be higher than objectively assessed knowledge.

## Introduction

Evidence-based practice (EBP) is a systematic approach where the current best available evidence from research is combined with clinical experience and patient preferences to make clinical decisions within a context and available resources [1]. As healthcare professionals are increasingly expected to use evidence from various sources to improve healthcare outcomes, there is a growing responsibility for educational programs to enhance students’ knowledge and skills in the EBP process and research methodology [1–4]. Knowledge of EBP terminology and research methodology are prerequisites to understand the concept of EBP, critically appraise research evidence, and integrate and apply evidence in clinical practice.

As educators implement EBP in curricula, they need reliable instruments to assess student knowledge, both formatively and summatively. In the second Sicily statement, Tilson et al. [5] presented the Classification Rubric for EBP Assessment Tools in Education (CREATE) framework, recommending a common taxonomy for tools assessing EBP learning. The framework refers to knowledge as “learner’s retention of facts and concepts about EBP”, and suggests assessments of EBP knowledge that evaluate a learner’s ability to define EBP concepts, describe level of evidence, or list basic principles of EBP [5].

Various instruments have been used to assess EBP knowledge among healthcare professionals [6–9]. Few tools have been validated for use among undergraduate students. Cardoso et al. [10] have published a protocol for a systematic review that aims to identify and assess properties of instruments for measuring knowledge, attitudes and skills in EBP among undergraduate nursing students. However, at the present time there are no systematic reviews of instruments used to assess EBP knowledge among healthcare students across disciplines. Typically, self-report scales that assess the steps of the EBP model (ask, acquire, appraise and apply) [11–13] or the understanding of common research terms [14] have been used to evaluate self-perceived (i.e. subjective) EBP knowledge. Objective knowledge has been evaluated with questionnaires including multiple-choice questions [15–17], or clinical scenario tasks with subsequent dichotomous [18, 19] or open-ended [20, 21] questions. Self-report instruments have advantages such as simple administration, low costs and greater feasibility. Evidence from other fields shows that self-report of skills and abilities correspond poorly to objective performance [22, 23].

Agreement between self-reported and objectively measured knowledge of EBP has not been widely investigated. Few studies report correlations between self-reported and objectively measured competence in critical appraisal and EBP terminology among undergraduate medical students [24], physicians [25], allied healthcare professionals [26] and nurses [27]. Other studies report only on separate results for the two outcome measures [28–32]. Whether self-rating scales in the field of EBP accurately reflect objective knowledge levels is largely unstudied, particularly among healthcare students. The aim of this study was to examine agreement between self-reported and objectively assessed knowledge of EBP terminology among healthcare students. A secondary objective was to explore agreement among students with different levels of EBP exposure.

## Materials and methods

We performed a cross-sectional study among students from various healthcare disciplines in one Norwegian University College and nursing students from one Canadian University, during winter 2016/2017.

### Setting

EBP is a national priority in Norwegian educational healthcare programs [33, 34] and there has been an increase in teaching and learning of EBP during the past decade. Nonetheless, at the time of data collection, EBP was not fully integrated in the curricula of the Norwegian University College and EBP exposure varied between programs. At the bachelor’s and master’s level all programs pursued competencies in EBP and research methodology, but the level and extent differed between programs (Table 1).

**Table 1:** Teaching of EBP critical appraisal skills and research methodology for bachelor and master students.

	Length of programs		Stand-alone course in EBP and/or research methodology	Teaching of EBP critical appraisal skills and/or research methodology	Evaluation in EBP critical appraisal skills and/or research methodology
	Semesters (Years)	Full/ Part-time	Courses (No. courses, total credit points, semester taught)	Semester	Formative (F), Summative (S)
**NORWAY**					
Bachelor in Nursing	6 (3)	F	EBP (1, 5 ECTS*, 4)	2, 4, 6	F
Bachelor in Occupational Therapy	6 (3)	F	No	3, 5, 6	F
Bachelor in Physiotherapy	6 (3)	F	No	1, 3, 4, 5, 6	F
Bachelor in Radiography	6 (3)	F	Research methodology (1, 5 ECTS, 5)	4, 5, 6	F
Master in Clinical Nursing specializing in anesthetics, surgical, intensive care, pediatric nursing	5 (2.5)	F/P**	EBP and research methodology (1, 15 ECTS, 3)	3, 4, 5	F
Master in Clinical Nursing specializing in diabetes, cardiac, public health nurse	6 (4)	P	EBP and research methodology (2, 25 ECTS, 1, 4)	1, 4, 5, 6	F, S
Master in EBP in Healthcare	8 (4)	P	EBP and research methodology (5, 75 ECTS, 1–5)	1, 2, 3, 5–8	F, S
**CANADA**					
Bachelor of Science in Nursing	8 (4)	F	Research methodology (1, 3 credits***, 8)	1–8	F, S
Master of Science in Nursing Course Based Primary Health Care Nurse Practitioner	6 (2)	F/P	EBP and research methodology (1, 3 credits, 1)	1, 2	F, S

*ECTS = European Credit Transfer and accumulation System. One credit corresponds to 25–30 hours of work.

**First 3 semesters (90 ECTS) were full-time and last two semesters (30 ECTS) were part-time studies.

***A credit is roughly equivalent to one lecture-hour per week for one term or two hours of laboratories or seminars per week for one term

In Canada, the consideration of research evidence in practice decisions is an increasingly part of individual standards of practice [35]. The curriculum of the Canadian University had included the teaching and learning of EBP for two decades. As of 2014, the bachelor’s of science in nursing program had EBP integrated through all four years in theory and clinical courses, supported with e-learning resources and summative assessments. At the master’s level, the students took a stand-alone one-semester course in EBP and research methodology, with reinforcement of this content in a subsequent course.

In Norway, the exposure of EBP terminology, critical appraisal skills and research methodology in teaching and learning was in general less for students at the bachelor’s as compared to the master’s level. In Canada, the exposure throughout the bachelor’s program may be similar to the exposure of the master’s students, but the master’s student experience was much more concentrated in one course. In this study, we have considered EBP exposure as higher among Norwegian master’s students and all Canadian students than among Norwegian bachelor’s students.

### Participants and data collection

Eligible participants from Norway (n = 336) were students at one University College and comprised final (3rd) year bachelor in nursing, occupational therapy, physiotherapy and radiography, as well as 2nd year master of clinical nursing specializing in anesthetics, surgical or intensive care nursing, 3rd year master of clinical nursing specializing in diabetes, cardiac or public health nursing, and 2nd and 4th year master of EBP in healthcare (Table 1). Eligible participants from Canada (n = 154) were 3rd year bachelor of science in nursing and 1st year master of science in nursing course based primary health care nurse practitioner students from one University.

We collected data in classrooms after teaching sessions. The class sessions varied in content and did not necessarily include teaching of EBP or research methodology. The Norwegian students received information about the study on their online learning platform two days before data collection, while the Canadian students were informed in the classrooms. The students were asked to complete a paper-based or electronic questionnaire that contained 18 questions related to their understanding of terms associated with EBP and research, and six open-ended questions where they were to elaborate on their understanding of a subset of the terms. Students answered and returned the self-reported part of the questionnaire before they received the open-ended questions. Students who preferred the electronic version used a link to the questionnaire from their online learning platform. The Norwegian students received a food voucher for dinner in the school cafeteria, as a token of appreciation.

### Measurement

The questionnaire consisted of demographic characteristics, 17 self-report questions from the Evidence-Based Practice Profile (EBP^2^) Terminology domain [14], one self-report question of how to understand the term ‘evidence-based practice’ and six open-ended questions formulated as “What does XX mean, in your own words, AND how would you describe it to a fellow student?”.

The EBP^2^ is a self-report trans-professional questionnaire that examines self-perceived EBP knowledge, attitude and behaviour. It consists of five domains (Relevance, Terminology, Confidence, Practice and Sympathy), where the EBP^2^ Terminology domain (17 items) examines knowledge related to the understanding of common research terms. EBP^2^ has previously been described with acceptable reliability and validity measures among Australian students and professionals across health disciplines [14]. The questionnaire has been translated into Norwegian, cross-culturally adapted and validated among Norwegian bachelor students and healthcare professionals from various disciplines. In the Norwegian version, the EBP^2^ Terminology domain was found reliable, valid and responsive to change [36].

**Fig 1:**
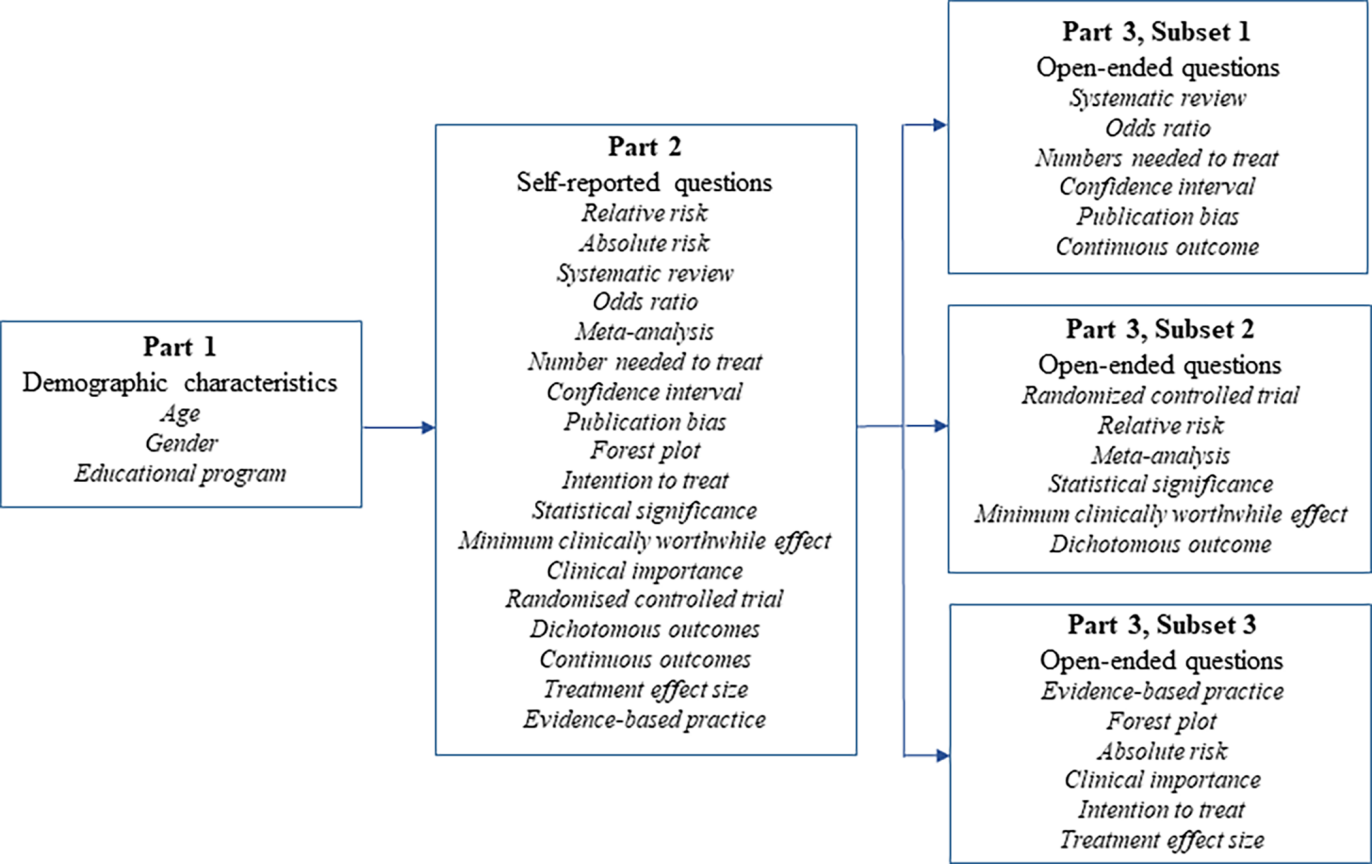
Questionnaire items, self-report and subsets of open-ended questions.

Specifically, the applied questionnaire consisted of three parts. Part 1 assessed demographic characteristics, including gender, age, educational program and educational institution. Part 2 examined self-reported knowledge and contained 18 items, whereof 17 originated from the EBP^2^ Terminology domain. In this part, participants rated their self-perceived understanding on a 5-point Likert scale, where 1 = “never heard the term”, 2 = “have heard it, but don’t understand”, 3 = “have some understanding”, 4 = “understand quite well” and 5 = “understand and could explain to others”. Part 3 examined objective knowledge, as assessed and rated by a rubric, and contained open-ended short answer questions derived from Part 2. To limit the time needed to complete the questionnaire, each participant was asked a subset of six open-ended questions. Thus, all 18 items were divided into three subsets (Fig 1), and each student received a subset chosen at random. There were a total of three question subsets, therefore agreement measures for each question were calculated on approximately a third of the total number of participants.

To assess the answers of the open-ended questions, we developed a five-level scoring rubric in close collaboration with experts in EBP from McMaster University (DC and JY). The scoring of the open-ended answers related to the 1–5 levels in the self-rating section of Part 2, with values from 1 “never heard the term” to 5 “understand and could explain to others”.

We performed a pilot study during spring term 2016 to test the understanding and interpretation of the scoring rubric, as well as the administration of the paper-based and the electronic version of the questionnaire. The pilot was performed among final-year Norwegian bachelor’s students in nursing and allied healthcare (n = 49) and recently graduated master’s students in EBP (n = 19). Two raters (AKS and DC) individually scored the answers, met at two occasions and discussed differences in scorings between raters. Adjustments in the scoring rubric to clarify wording and distinctions between levels of grading were made, and two decision rules to the final scoring rubric (available upon request from first author) were added. Finally, the two raters individually scored the remaining pilot questionnaires (n = 53). Interrater agreement with linear weighted kappa (K_lw_) demonstrated an almost perfect agreement between raters (K_lw_ = 0.81).

In the current study, one rater (AKS) scored the Norwegian questionnaires and one rater (DC) scored the Canadian questionnaires. All open-ended questions that were left blank (not answered) were scored as 1 “never heard the term”. Respondents who did not answer any questions in part three were excluded from analysis.

### Statistical analysis

A power analysis informed that 78 students were needed to estimate agreement between self-perceived and assessed open-ended answers (kappa value of 0.8 with a lower confidence limit of 0.7). Since a student would give open-ended answers to a third of the items only, a total of 234 students needed to be enrolled. The significance level was set to 0.05. The sample size calculations were performed using the CI5Cats function in the kappaSize package in R [37].

Descriptive analyses were applied for demographic characteristics. Mean (M) and standard deviation (SD) were reported to describe the scores of the self-reported and assessed open-ended items. Due to the ordinal measurement level, weighted kappa was used to estimate interrater agreement between self-reported knowledge and assessed open-ended answers for each research term. To provide complementary information on the distribution of disagreement, we calculated both quadratic (K_qw_) and linear weighted kappa (K_lw_). Furthermore, overall summary score was calculated for the EBP^2^ Terminology domain by summing the scores of the 17 items within the domain. We used the intraclass correlation coefficient for absolute agreement (ICC) to estimate overall agreement for the EBP^2^ Terminology domain.

Mean differences between self-reported and assessed open-ended items were estimated with paired t-test. We used independent sample t-test to analyze differences in mean self-reported EBP^2^ Terminology domain scores by EBP exposure.

P-values less than 0.05 indicated statistical significance. Kappa values were considered poor if < 0, slight if 0–0.20, fair if 0.21–0.40, moderate if 0.41–0.60, substantial if 0.61–0.80 and almost perfect if 0.81–1.0 [38].

The statistical software IBM SPSS Statistics version 22 [39] and *R* [40] were used for the statistical analyses.

### Ethics

The Norwegian Social Science Data Services (NSD) (Reference number 49132), and The Hamilton Integrated Research Ethics Board (Project number 2463) approved the study.

The survey was voluntary. In Norway, consent for participation was completion and return of the questionnaire. In Canada, the students signed a consent for participation. Data was analysed and stored in the research server at the Western Norway University of Applied Sciences.

### User involvement

A user panel of four Norwegian bachelor students, one from each health discipline, constituted the user involvement in this study. The users assisted in the collection of data by finding appropriate times for data collection and encouraging peer students to participate in the study. They also participated in the interpretation and discussion of the results. The user panel met on three occasions, to receive information about the study, plan the data collection and discuss results. E-mail correspondence was used between meetings.

## Results

Of all eligible students, 291 (59%) answered the questionnaire. The response rate was higher in Norway (70%) than in Canada (37%). Four students were excluded, as they had not answered part three of the questionnaire, allowing 287 respondents to be included in the analysis.

Our sample included bachelor students in nursing (53%) and allied health professions (29%), and master students in nursing (10%) and in evidence-based practice (8%) (Table 2). The mean age was 26.4 (SD = 8.4) years and the majority were females (87%). The sample consisted of a higher proportion of Norwegian (80%) than Canadian (20%) students.

**Table 2:** Characteristics of participants.

	Total (n = 291)	Norway (n = 234)	Canada (n = 57)
	n (%)	n (%)	n (%)
**Gender**			
Female	253 (87)	201 (86)	52 (91)
Male	33 (11)	28 (12)	5 (9)
Missing	5 (2)	5 (2)	0
**Educational program**			
Bachelor in nursing	155 (53)	105 (45)	50 (88)
Bachelor in occupational therapy	23 (8)	23 (10)	
Bachelor in physiotherapy	43 (15)	43 (18)	
Bachelor in radiography	16 (6)	16 (7)	
Master in nursing	30 (10)	23 (10)	7 (12)
Master in evidence-based practice	24 (8)	24 (10)	
**Age**			
N	252	195	57
Mean (SD)	26.4 (8.4)	27.8 (8.8)	21.6 (4.4)
Min–Max	19–56	21–56	19–51

The mean self-reported scores for the terms included in the EBP^2^ Terminology domain varied from 1.99 (‘forest plot’) to 4.20 (‘systematic review’) (Table 3). The self-reported mean score for the item ‘evidence-based practice’ was 4.33, (SD = 0.8). The overall self-reported mean EBP^2^ Terminology score was 3.02 (SD = 0.87).

**Table 3:** Agreement values for the EBP^2^ terminology domain and research terms.

	Mean scores (SD)				Weighted kappa
Items	n	Self-reported	n	Assessed	Quadratic (95% CI)
**EBP**^**2**^ **Terminology domain**					
** Forest plot**	89	1.99 (1.28)	89	1.46 (1.09	0.69 (0.55–0.83)
** Dichotomous outcome**	100	2.23 (1.56)	100	1.64 (1.10)	0.67 (0.55–0.79)
** Numbers needed to treat**	97	2.62 (1.36)	98	2.00 (1.32)	0.60 (0.46–0.73)
** Confidence interval**	98	2.87 (1.37)	98	1.86 (1.11)	0.50 (0.39–0.62)
** Continuous outcome**	98	2.61 (1.41)	98	1.49 (1.02)	0.39 (0.26–0.52)
** Meta-analysis**	100	3.25 (0.94)	100	1.95 (1.16)	0.30 (0.17–0.43)
** Treatment effect size**	89	2.88 (1.21)	89	1.80 (0.97)	0.29 (0.17–0.41)
** Relative risk**	100	3.09 (1.17)	99	1.72 (1.02)	0.22 (0.12–0.32)
** Statistical significance**	100	3.61 (1.20)	100	2.20 (1.16)	0.21 (0.09–0.33)
** Intention to treat**	89	2.74 (1.28)	89	1.28 (0.84)	0.18 (0.07–0.30)
** Odds ratio**	98	2.52 (0.94)	98	1.46 (0.68)	0.17 (0.07–0.27)
** Randomized controlled trial**	100	4.14 (0.99)	100	2.31 (1.14)	0.16 (0.08–0.24)
** Publication bias**	98	3.18 (1.42)	98	1.23 (0.73)	0.09 (0.02–0.17)
** Systematic review**	96	4.20 (0.82)	98	2.12 (0.84)	0.08 (0.03–0.12)
** Min clinically worthwhile effect**	100	2.57 (1.24)	100	1.29 (0.72)	0.07 (-0.02–0.17)
** Clinical importance**	89	3.89 (1.07)	89	1.63 (0.68)	0.06 (0.01–0.11)
** Absolute risk**	89	3.01 (1.07)	89	1.46 (0.88)	0.04 (-0.03–0.11)
**Evidence-based practice**	89	4.33 (0.80)	89	2.74 (1.03)	0.13 (0.04–0.22)

The assessed open-ended mean scores for the terms included in the EBP^2^ Terminology domain varied from 1.23 (‘publication bias’) to 2.31 (‘randomized controlled trial’) (Table 3). The assessed open-ended mean score for the item ‘evidence-based practice’ was 2.74 (SD = 1.0). The overall assessed open-ended mean score for EBP^2^ Terminology was 1.70 (SD = 0.68).

For all research terms, self-reported knowledge was higher than assessed (p<0.001). Still, we observed large variations in agreement values between self-reported and assessed open-ended items (Table 3). We found substantial agreement for the items ‘forest plot’ (K_qw_ = 0.69) and ‘dichotomous outcome’ (K_qw_ = 0.67), and moderate agreement for the items ‘numbers needed to treat’ (K_qw_ = 0.60) and ‘confidence interval’ (K_qw_ = 0.50). Moreover, we observed fair agreement for five items, and slight agreement for the remaining nine. Analysed with linear weighted kappa, agreement values were lower for all items. We found low overall agreement between the self-reported and objectively assessed open-ended items of the EBP^2^ Terminology domain (ICC = 0.29; 95% CI: -0.09–0.62).

Agreement measures were equal for high (ICC = 0.11; 95% CI: -0.07–0.33) and low (ICC = 0.11; 95% CI: -0.07–0.32) exposure of EBP. These findings were consistent with analyses performed for each question subset S1 Table. High exposed students had a significantly higher self-reported mean EBP^2^ Terminology score compared to that of low exposed students (MD = 1.19, p < 0.001) S2 Table.

## Discussion

In this study, we found overall low agreement between healthcare students’ self-reported and objectively assessed knowledge of EBP terminology, as rated by a rubric. However, agreement varied by research terms. We found substantial agreement for the research terms with the lowest self-reported mean scores and slight agreement for the research terms with highest self-reported mean scores. We observed no difference in agreement values for students with high or low EBP exposure. However, self-reported scores were on average higher for students with high EBP exposure than with low exposure.

To the best of our knowledge, few studies have previously made comparisons between self-reported and objectively assessed knowledge in the field of EBP knowledge. Previous studies assessing the relationship between self-reported and objective measured EBP knowledge have reported small to medium correlations between self-reported and objectively measured competence in critical appraisal among senior medical students [24] and health professionals [26]. Others have reported small, non-significant correlations between self-reported and objective measures of EBP knowledge among nurses [27] and physicians [25]. However, by reporting correlation coefficients, previous studies have reported the strength of a linear association between two variables, and not the agreement between them [41]. Direct comparisons of results should therefore be performed with caution.

Consistent with previous studies [24, 25, 32], our participants over-estimated their self-reported EBP knowledge. One factor influencing self-ratings may be social desirability bias. This mechanism, where respondents answer in a manner that would be viewed favorably, has also been seen in other fields of research, such as when self-reporting physical activity [23] and self-reporting height, weight and body mass index [42]. Another explanation may be that the students lacked the ability to judge their own knowledge and skills, maybe due to lack of internal yardstick or understanding of expectations. In a study of performance on social and intellectual tasks, Ehrlinger et al. [43] found that poor performers overestimated performance, and argue that incompetence may deprive us of insight regarding our deficits.

The students’ responses and the poor agreement we observed may also have a simpler explanation. Context and motivations for using EBP may influence assessments [5], and there is no reason to believe that this study is different. For instance, the motivation to recall knowledge and write down answers to the open-ended questions is a demanding task. Perhaps the students lacked motivation to write out the answers during the data collection period. As such, we have no way of telling whether the respondents could have demonstrated higher levels of understanding in their open-ended answers if they were able to verbally respond to the short answer questions, if their motivation was different, or if they were allowed to use the resources that they can use in real-life situations. Also, Zell and Krizan [22] argue that self-assessment for tasks that are familiar and have low complexity corresponds better than unfamiliar and high-complexity tasks.

We found overall low agreement between self-reported and objectively assessed knowledge in EBP terminology, but with large variations in agreement values between items. Highest agreement was found for the research terms with lowest self-reported mean scores. For example, for ‘forest plot’ most students answered 1 (“never heard the term”) on the self-report and “I don’t know” for the corresponding open-ended question. Conversely, for terms that students reported higher levels of knowledge, such as ‘evidence-based practice’, ‘systematic review’, and ‘randomized controlled trial’, we found high self-reported scores and slight agreement values. For these items, we observed large differences between responders and raters’ classifications, indicating that our responders may not have been as knowledgeable as they reported. However, it could also be argued that the higher agreement found for items with the lowest self-reported scores may not reflect a better understanding of own knowledge, but rather be ascribed to a floor effect limiting variation in self-reported and objectively assessed answers. Still, with additional evidence from other disciplines revealing poor correspondence between self-evaluations of abilities and objective performance measures [22, 23], we question whether self-reported knowledge of EBP terminology, as measured in the EBP^2^ Terminology domain, is a good proxy for objective knowledge of EBP.

Blanch-Hartigan [44] described that medical students’ ability to self-assess performance was more accurate later in medical school as compared to earlier in medical school. In our study, we conjectured that students with higher exposures of EBP would rate themselves higher on the self-reported EBP^2^ Terminology domain, obtain higher assessed scores on their open-ended answers, and have better agreement values than students with lower exposures of EBP. As hypothesized and previously described [14, 37], we found that the self-reported EBP^2^ Terminology domain discriminated between levels of EBP exposure. However, we found no differences in agreement values for students with different exposures of EBP.

### Limitations

The main limitation of this study was that the open-ended questions and scoring rubric had not been evaluated for reliability and validity. We attempted to overcome this limitation by ensuring that experts in EBP developed the rubric and adapted it to both settings before use. In addition, we performed a pilot in which we found an almost perfect agreement between raters.

At the time of data collection, EBP^2^ was the only questionnaire that examined knowledge related to EBP terminology among students across health disciplines. By applying the EBP^2^ Terminology domain, we have only assessed one part of the EBP^2^ questionnaire. Furthermore, EBP terminology is only one facet of EBP. By not assessing knowledge related to all steps of the EBP model (ask, acquire, appraise, apply or assess), we have examined a limited dimension of knowledge related to EBP.

We have no further information of our responders’ confidence and competence in EBP, apart from the knowledge of EBP terminology we assessed at this one point of time. We recognize that a convenience sample of students from two educational institutions in two different countries may have hampered generalizability of the study. Furthermore, there was heterogeneity among the Norwegian master students regarding EBP exposure, as a newly started master program had not integrated EBP to the same extent as the two other programs.

We included sufficient participants to analyze agreement between self-reported and objectively assessed knowledge. Due to the smaller sample size of master students and Canadian students, agreement values between levels of EBP exposure should be interpreted with caution. We did not want variations in resources to influence the answers, and our participants answered the questionnaire under similar conditions. By administering the questionnaire anonymously in classrooms, we excluded a large proportion of eligible students.

## Conclusion

We found overall low agreement between healthcare students self-reported and objectively assessed knowledge of EBP terminology. The self-reported EBP^2^ Terminology domain discriminated between levels of EBP exposure. As a measurement tool, the EBP^2^ Terminology scale may be useful to discriminate between levels of EBP exposure.

As a discriminatory tool for the purpose of academic promotion or clinical certification, users should be aware that self-ratings would be higher than objectively assessed knowledge.

## Supporting information

S1 Table Agreement values for EBP exposure, analyzed for subsets of open-ended questions. (DOCX)

S2 Table Self-reported EBP^2^ terminology domain scores and EBP exposure. (DOCX)

S1 File Data set. (ZIP)
