# Supplementary material for: Self-reported and objectively assessed knowledge of evidence-based practice terminology among healthcare students: A cross-sectional study
Source: PLoS One. 2018 Jul 12;13(7):e0200313. doi: 10.1371/journal.pone.0200313 (PMC6042753; doi:10.1371/journal.pone.0200313)
Supplement: S1 Table — (DOCX) [file pone.0200313.s001.docx]

# Supporting Information

**S1 Table. Agreement values for EBP exposure, analyzed for subsets of open-ended questions.**

| **Subset** | **Exposure*** | **n** | **ICC** (95% CI).** |
| --- | --- | --- | --- |
| **1** | All | 95 | 0.28 (-0.09 – 0.62) |
|  | Low | 60 | 0.15 (-0.07 – 0.43) |
|  | High | 35 | 0.16 (-0.07 – 0.46) |
| **2** | All | 98 | 0.27 (-0.09 – 0.60) |
|  | Low | 64 | 0.11 (-0.07 – 0.34) |
|  | High | 34 | 0.07 (-0.07 – 0.26) |
| **3** | All | 87 | 0.17 (-0.07 – 0.47) |
|  | Low | 62 | 0.05 (-0.05 – 0.20) |
|  | High | 25 | 0.06 (-0.05 – 0.26) |

**Low exposure = Bachelor’s students from Norway; High exposure = Master’s students from Norway and undergraduate and master’s students from Canada*

***ICC for absolute agreement*
