# Supplementary material for: Self-reported and objectively assessed knowledge of evidence-based practice terminology among healthcare students: A cross-sectional study
Source: PLoS One. 2018 Jul 12;13(7):e0200313. doi: 10.1371/journal.pone.0200313 (PMC6042753; doi:10.1371/journal.pone.0200313)
Supplement: S2 Table — (DOCX) [file pone.0200313.s002.docx]

**S2 Table.** Self-reported EBP^2^ Terminology domain scores and EBP exposure.

|  | **EBP^2^ Terminology domain** | | | |
| --- | --- | --- | --- | --- |
| **EBP exposure*** | **n** | **Mean (SD)** | **Mean Difference (95%CI)** | **P value** |
| Low | 187 | 2.58 (0.60) |  |  |
| High | 104 | 3.77 (0.58) | 1.19 (1.04 – 1.33) | <0.001 |

**Low exposure = Bachelor’s students from Norway; High exposure = Master’s students from Norway and undergraduate and master’s students from Canada*
